# Management of Anti-melanoma Differentiation-Associated Gene 5 (Anti-MDA5)-Positive Dermatomyositis in an Acute Rehabilitation Center: A Case Report

**DOI:** 10.7759/cureus.27791

**Published:** 2022-08-08

**Authors:** Roi F Medina, John Jahan

**Affiliations:** 1 Department of Physical Medicine and Rehabilitation, Lake Erie College of Osteopathic Medicine, Bradenton, USA; 2 Department of Physical Medicine and Rehabilitation, Sharp Memorial Hospital, San Diego, USA

**Keywords:** anti-mda5, melanoma differentiation-associated gene 5, myositis-specific antibody, autoantibodies, mda5, comprehensive rehabilitation, acute rehabilitation, idiopathic inflammatory myopathies, myositis, dermatomyositis

## Abstract

Dermatomyositis is an immune-mediated myopathy predominantly characterized by skin and musculoskeletal manifestations. Although often described in the context of its pathognomonic cutaneous features, dermatomyositis may have variable presentations depending on the subtype. In this report, we present a case of anti-melanoma differentiation-associated gene 5 (anti-MDA5)-positive dermatomyositis that initially presented with respiratory symptoms. An appropriate diagnosis and comprehensive interdisciplinary rehabilitation led to a significant improvement in functional status and a better overall prognosis for the patient.

## Introduction

Dermatomyositis is an idiopathic, immune-mediated myopathy that is predominantly characterized by skin and musculoskeletal manifestations [1]. It is a relatively uncommon disorder that has several subtypes and occurs in both juvenile and adult forms. The estimated annual incidence of all subtypes of dermatomyositis is one in 100,000 persons, with a higher incidence found in females than males [2]. Although the precise pathogenic mechanism of dermatomyositis is incompletely understood, early models suggest dermatomyositis muscle fiber injury resulting from antibody- and complement-mediated microangiopathy [1,3]. This is in contrast to more recent studies involving the use of genomic technologies on dermatomyositis skin and muscle samples that suggest type 1 interferon being involved in the pathogenesis and ultimately causing tissue injury [4-6].

Although dermatomyositis has various clinical manifestations, pathognomonic cutaneous features include Gottron papules, heliotrope rash, and shawl signs. Other characteristic cutaneous findings include facial and photo-distributed erythema, poikiloderma, nailfold and scalp abnormalities, and calcinosis cutis [7]. These cutaneous manifestations often precede or accompany muscle weakness, which is often described as symmetric, proximal, and insidious in development [8]. With disease progression, patients also experience esophageal, pulmonary, and cardiac symptoms, along with an increased risk of underlying malignancy [9]. Here, we present the management and progression of a patient admitted into an acute rehabilitation center with a diagnosis of anti-melanoma differentiation-associated gene 5 (anti-MDA5)-positive dermatomyositis.

## Case presentation

Approximately a month after initially experiencing symptoms of bilateral ocular edema and erythema, a 42-year-old female presented to her primary care physician. Since the initial onset of her symptoms, she also began experiencing coughs, fatigue, and oral ulcers. She was initially diagnosed with what was suspected to be bacterial pneumonia and started on a course of antibiotics. A week later, after experiencing minimal improvement in her symptoms along with worsening fevers, she was admitted to the hospital. At this time, physical examination revealed an erythematous rash on her palms, knuckles, elbows, and chest. An inflammatory workup at the time revealed a positive antinuclear antibody (ANA) with titer 1:320 and a homogenous pattern. Further workup revealed positive anti-Sjögren's syndrome A antibody (SS-A) with titer 46.46. The patient opted to leave the day after admission but returned to the emergency department a week later with worsening symptoms and new onset oral thrush and progressive dysphagia. She was hospitalized with symptoms consistent with dermatomyositis. She received a course of prednisone 60 mg and was discharged six days later. Twelve days after discharge, she returned to the emergency department with generalized weakness, fatigue, worsening oral thrush, dysphagia, and worsening rash overlying the chest. She was admitted to the hospital for the third time and diagnosed with adult dermatomyositis with lung involvement after undergoing chest computed tomography (CT) that revealed diffuse ground-glass opacities. Additional workup at this time revealed elevations in aldolase and creatinine phosphokinase (CPK). Lower extremity magnetic resonance imaging (MRI) was also performed and revealed muscle edema. At this time, she was seen by a rheumatologist and prescribed Rituxan, high-dose steroids, and eventually a course of intravenous immunoglobulin (IVIG). She also began occupational therapy, physical therapy, and speech therapy sessions. However, due to ongoing muscle weakness and persistent dysphagia, she was referred to an acute rehabilitation center three weeks after admission. The patient’s initial functional status at the time of admission into the acute rehabilitation center can be seen below (Tables 1-3).

**Table 1:** Physical therapy (complete assessment at admission limited by generalized weakness and decreased activity tolerance)

Bed mobility/transfers		
Parameter	Result (admission)	Result (discharge)
Rolling	Moderate assist	Independent
Supine-to-sit	Moderate assist	Independent
Sit-to-supine	Moderate assist	Independent
Sit-to-stand	Moderate assist	Supervised
Stand-to-sit	Moderate assist	Supervised
Transfers	Moderate assist	Supervised
Ambulation/gait		
Parameter	Result (admission)	Result (discharge)
Assistive device	Wheelchair	Front-wheeled walker
Ambulation (level surfaces)	N/A	200 ft - supervised
Ambulation (uneven surfaces)	N/A	50 ft - supervised
Curbs	N/A	Supervised
Stairs	N/A	5 stairs – contact-guard assist
Ramp	N/A	Supervised

**Table 2:** Occupational therapy

Parameter	Result (admission)	Result (discharge)
Oral hygiene	Moderate assist	Independent
Toileting hygiene	Max assist	Independent
Feeding	Total assist via percutaneous endoscopic gastrostomy (PEG) tube	Independent
Dressing (upper extremity)	Moderate-max assist	Independent
Dressing (lower extremity)	Max assist	Independent
Bathing (upper extremity)	Max assist	Independent
Bathing (lower extremity)	Max assist	Independent
Putting on, taking off footwear	Max assist	Independent
Toilet transfer	Moderate assist	Independent

**Table 3:** Speech therapy

Parameter	Result (admission)	Result (discharge)
Clinical swallowing	Clear-liquid diet	All liquids, chopped solids
Communication	Moderate-severe dysarthria w/ dysphonia. Reduced attention and memory of complex information, and prolonged information processing. Moderate cognitive-communication deficits	Mild-moderate dysarthria and intelligible for sentence-level information and brief conversations. Able to recall semi-complex information. Mild cognitive-communication deficits

This patient, who was completely independent with a normal prior level of function, presented to the acute rehabilitation center with primary rehabilitation diagnoses of debility, dysphagia, generalized weakness, impaired mobility, impaired self-care and toileting, and incontinence of bowel. Past medical history included major depressive disorder and generalized anxiety disorder. Family history was not significant for autoimmune disorders. The patient had no history of tobacco or alcohol use. At admission, she had minimal skin findings with the exception of a few erythematous papules on the volar aspect of her fingers. Blood pressure and pulse were 95/63 mmHg and 88 beats/minute, respectively. Respiratory rate was 18 breaths/minute with oxygenation saturation of 100% on room air. Musculoskeletal examination revealed mild upper extremity weakness and moderate lower extremity weakness with normal reflexes. Baseline blood tests demonstrated hemoglobin at 9.5 g/dL (normal: 12.0-16.0 g/dL), hematocrit at 27.2% (normal: 36-46%), white blood cell count (WBC) of 6,300/mm^3^ (normal: 4,500-11,000/mm^3^), platelet count of 253,000/mm^3 ^(normal: 150,000-400,00/mm^3^), sodium at 133 mEq/L (normal: 136-146 mEq/L), potassium at 3.7 mEq/L (normal: 3.5-5.0 mEq/L), alanine aminotransferase (ALT) at 100 IU/L (normal: 5-45 IU/L), aspartate aminotransferase (AST) at 84 IU/L (normal: 5-45 IU/L), albumin at 3.0 g/dL (normal: 3.5-5.5 g/dL), and CPK at 197 mcg/L (normal: 10-120 mcg/L). At this time, she was receiving prednisone 60 mg per her gastrostomy tube daily and applying prednisone ophthalmic drops in each eye four times daily as needed for ongoing ocular erythema and edema. The patient was started on a comprehensive interdisciplinary rehabilitation program involving physical, occupational, speech-language, nutrition, and recreational therapies.

**Figure 1:**
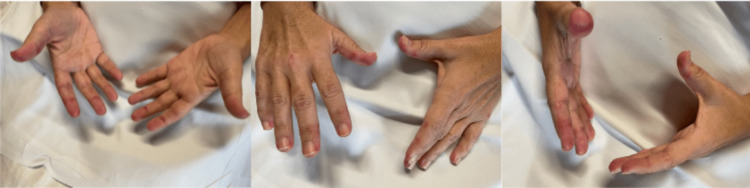
Gottron papules in a 42-year-old female with dermatomyositis

Over the course of the next three weeks, the patient continued with daily therapies and demonstrated progressive improvement in overall function without any complications. At the advice of her rheumatologist, daily prednisone was first tapered down to 50 mg daily after eight days before eventually tapering to 40 mg after an additional 10 days. The patient was also started on azathioprine 50 mg once daily at the end of the second week with an increase to twice daily after the first 14 days. At the beginning of the fourth week, when the patient’s prednisone was tapered down to 40 mg, she initially experienced relapsing Gottron papules bilaterally (Figure 1). No medication changes were made and the patient showed noticeable improvement in these symptoms over time. At 31 days in rehabilitation, the patient met her short-term goals and was discharged. The patient’s functional status at the time of discharge is shown in Tables 1-3. Her discharge plan included continuing daily prednisone and azathioprine, as well as following up with her rheumatologist and primary care provider. Home health therapy was also arranged with the hope of maintaining strength and mobility and continuing functional improvement.

## Discussion

**Figure 2:**
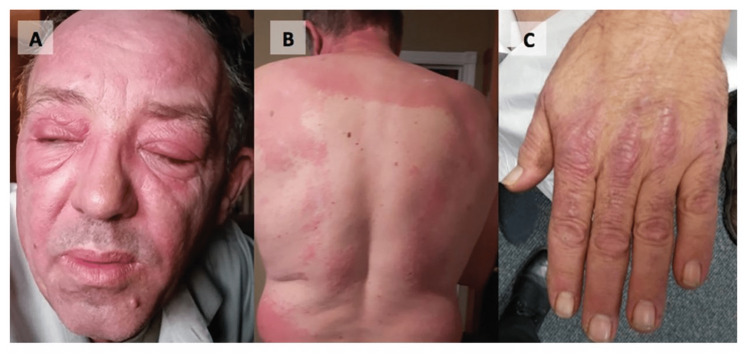
(A) Heliotrope rash, (B) shawl sign, and (C) Gottron papules in a 53-year-old male with dermatomyositis Images in this figure have no relation to the subject of this case report. Images were obtained from [10]. Used with permission.

The diagnostic criteria of Bohan and Peter [10] established in 1975 are widely used but have limitations as each criterion lacks a clear definition, leading to difficulties with deciphering between different forms of myopathies. The European League Against Rheumatism (EULAR)/American College of Rheumatology (ACR) classification criteria for adult and juvenile idiopathic inflammatory myopathies provide more up-to-date criteria that are based on the age of onset, presence of muscle weakness, characteristic skin manifestations (Figure 2) [11], abnormal laboratory measurements, muscle biopsy features, and the presence of other clinical manifestations including dysphagia and esophageal dysmotility [12]. A patient classified with idiopathic inflammatory myopathy by the EULAR/ACR criteria is also subclassified using the above-mentioned variables [12]. Diagnostic workup of dermatomyositis may also involve the detection of myositis-specific autoantibodies that are associated with unique clinical phenotypes [13]. Myositis-specific autoantibodies may be present in up to 45-85% of patients [14]. Although there is limited evidence of these autoantibodies being directly involved in the pathogenesis of the disease, clinicians may use them to guide them through screening and treatment protocols.

Our patient presented with many of the diagnostic features and was treated accordingly. On the myositis screen, there was the presence of anti-MDA5 autoantibodies. Anti-MDA5 dermatomyositis was first described in 2009 [15] and is of variable frequency, ranging from 7% to 25% of adult dermatomyositis cases [16,17]. Along with distinctive mucocutaneous symptoms, it has an elevated risk of interstitial lung disease [18]. The diagnosis is often overlooked or delayed, as the initial presentation with predominant lung involvement may direct clinicians toward more common causes of interstitial lung disease, as seen in this case.

Until the early 1990s, patients diagnosed with inflammatory myopathies were generally discouraged to partake in physical activity as a form of therapy due to concerns of exacerbating muscle inflammation. Alexanderson and Lundberg reviewed studies that highlight the safety and potential therapeutic benefits of active exercise including muscular and aerobic training [19]. Although they concluded that active exercise adapted to disease activity and patient tolerance may be recommended in the rehabilitation of patients with inflammatory myopathies, it was evident that the studies were few and sample sizes were small [19]. A more recent randomized control trial involving 21 patients with polymyositis was conducted to assess the post-rehabilitation improvements in function and quality of life [20]. The intervention group underwent a four-week, hospital-based rehabilitation program followed by a home-based, personalized program. Compared to the control group, in which the participants received only outpatient physiotherapy, the intervention group experienced significant improvement in disability index score, as well as multiple quality-of-life dimensions [20].

## Conclusions

It is evident that there is a role for physical exercise in the functional recovery of patients diagnosed with inflammatory myopathy. However, published studies assessing the specific role of a comprehensive interdisciplinary rehabilitation program in the functional recovery of patients with dermatomyositis are scarce. Given the variable presentation, poor prognosis, and potentially fatal course of anti-MDA5 dermatomyositis, timely identification and prompt intervention are crucial to reduce mortality rates and accelerate recovery. This case report emphasizes this point but also provides a glimpse into the functional benefits that may potentially be seen in a larger, multi-center study involving patients with dermatomyositis undergoing comprehensive rehabilitation.
